# Outpatient physical therapy population has been aging faster than the general population: a total population register-based study

**DOI:** 10.1186/s12913-021-06738-0

**Published:** 2021-07-18

**Authors:** Solveig A. Arnadottir, Brynjolfur Gauti Jonsson

**Affiliations:** 1 Department of Physical Therapy, Faculty of Medicine, School of Health Sciences, University of Iceland, Stapi v/Hringbraut, 102 Reykjavik, Iceland; 2 Statistical Consulting Center, School of Health Sciences, University of Iceland, Reykjavik, Iceland

**Keywords:** Population Dynamics, Aging, Health services, Outpatients, Registries, Big data

## Abstract

**Background:**

The Icelandic population is aging like other populations in the world, the life expectancy is high, and the national focus is to help people to age in their own homes. The objectives of this research was to describe 17 years of demographic changes among outpatient physical therapy (OPT) clients and to determine if these changes reflect aging in the total population.

**Methods:**

Data was obtained from a national registry with information on all OPT clients reimbursed by Icelandic Health Insurance from 1999 to 2015, and general population data from the Statistics Iceland registry covering the same 17 years. Simple counts, proportions, Rate Ratios (RR) and 95 % Confidence Intervals (CI) were used to describe and compare the two time-points (1999 and 2015) in both populations, and regression analyses were used to estimate linear changes for each of these 17 years.

**Results:**

Comparing the endpoints of the 17-year period, the proportion of older adults within the total OPT clientele increased by 23 % (from 18.3 % to 1999 to 23.5 % in 2015; RR 1.23; 95 %CI 1.19–1.27).) while in the general Icelandic population, the proportion of older adults increased by 15 % (from 11.6 % to 1999 to 13.5 % in 2015; RR 1.15; 95 % CI 1.1–1.21). For each of these 17 years, there was an overall 5 % yearly increase in the rate of older adults from the general older Icelandic population who used an OPT (accounting for population aging), and an overall 3.5 % yearly increase in the proportional contribution of older adults to the total OPT clientele. Adjusting for sex and older age group revealed that this increase in rate and proportion was most pronounced among ≥ 85-year-old men.

**Conclusions:**

This case of Iceland is an example of how health-related and population-based registers may potentially be used to routinely inform and facilitate optimal planning of future health care services for older adults.

## Introduction

The concept of “Retooling the Health Care Workforce for Aging Populations” was introduced in 2008 in a report from the Institute of Medicine in the USA [1]. The message of the report spread to other countries and to specific professional groups which have used the report’s content to advocate shared responsibility among professionals to facilitate optimal aging. In 2014, a physical therapy (PT) version of this “re-tooling wave” was published in the article “Building the Physical Therapy Workforce for an Aging America”[2]. Although these two papers were directed toward North American reality [1, 2], the message for physical therapists has been universal: (1) Older adults will comprise the largest percentage of PT clients across most practice settings, and (2) physical therapists who specialize in geriatrics are far too few to meet older adults’ needs. Hence, leaders in the field of geriatric PT have developed and published guidelines on essential competencies in the care of older adults and standards of practice aimed at all physical therapists who at any point work with older adults [3–5].

A call for increased emphasis on aging within the health care workforce is based on the fact that in aging populations, trends in disability and poor health are of particular concern [6, 7]. Different theories on population health change have been put forward to shed light on such trends. The most pessimistic theory is on *expansion of morbidity* where it is argued that increased length of life means that more years are spent in poor health and with disabilities, as people are living longer with chronic medical conditions, and with an increasing burden of age-related diseases [8]. A more optimistic view is presented in a theory on *compression of morbidity* where increased longevity is linked to fewer medical conditions and less disability due to improvements in preventive approaches and interventions [9]. A theory of *dynamic equilibrium*, however, relates increased longevity to a decreased prevalence of severe disabilities and medical conditions, although there is an increase in minor disabilities and medical conditions [10]. Data from across the world reveal that the trend in health conditions and disabilities varies between countries [7], with all three theories being supported in certain contexts. Despite this variability the following appears to be universal; older people are at the highest risk of all to have poor health and disabilities [11, 12], and all over the world there is an absolute increase in this at-risk population [11, 13]. Therefore, even the most optimistic prognoses foresee an absolute increase in the resources needed to maximize health and well-being in old age [6, 14].

Now, more than a decade after the 2008 Institute of Medicine report [1] it is important to look back and gather evidence on how demographic changes have been influencing health care practices and workforce. In Iceland, as an example, over 50 % of physical therapists are estimated to work in outpatient clinics where the focus has traditionally not been on aging or geriatrics. Yet the Icelandic population is aging (like other populations in the world), both men and women have high life expectancy [15, 16], and aging in place is generally favored by older adults and policy-makers [17]. From the perspective of population aging and increased emphasis on aging in place, outpatient physical therapy (OPT) may be an increasingly valuable resource to facilitate functioning and health among community-dwelling older adults, who are not in need of acute care hospitalization or inpatient rehabilitation. To date, however, research is needed on if and how demographic changes have been influencing OPT practices.

This research was built on 17 years (1999–2015) of complete data from two national electronic registers. The overall aim was to explore whether the OPT clientele reflects changes in population demographics (age, sex), which would support a routine use of demography to inform the planning of future OPT services. More specifically, the research objectives were to explore, over 17-year period: (1) changes in the proportion of older adults (≥ 65 years old) among OPT clients and to compare it to demographic changes in the general Icelandic population, and (2) the yearly rate for the number of older adults from the general population seeking OPT services, accounting for population aging. Both objectives included subgroup analyses for three age groups of older adults (65–74, 75–84, ≥ 85 years), and for sex (men, women).

## Methods

### Research design

This was an observational, longitudinal research on register-based data from two Icelandic national electronic registries, covering a 17-year period from 1999 to 2015.

### Setting

The context of the research is Iceland, where the health care system is centralized, and publicly financed through an annual national budget [17].

#### Outpatient physical therapy (OPT)

In 2015, approximately 550 physical therapists were practicing in Iceland [18]. No legislation prevents private practice, which is a context where over half of all physical therapists are estimated to work. In a 2013 survey of the total population of full members of the Icelandic Physical Therapy Association, 52.5 % of the respondents described their facility as a private outpatient practice [19]. These physical therapists provide their services (individual and group therapy) in special OPT clinics outside of institutional settings (hospitals, rehabilitation centers, nursing homes, etc.) to people of all ages who live in the community. Physical therapists in Iceland are autonomous practitioners to whom patients or clients have direct access, offering an entry point into the health care system [18].

#### Geriatric certified specialists

Icelandic physical therapists can become clinical specialists, certified by the Directorate of Health (law) [20]. In 2015, 9.6 % (53/550) of physical therapists had received a specialist certification, three out of these 53 specialized in geriatric PT and none of them worked in OPT [21].

#### Health insurance

In Iceland, health care is publicly funded, meaning that residents are insured by the state, with equal access for everyone [22]. Everyone who has been a legal resident in Iceland for six months automatically becomes a member of the Icelandic social insurance system, regardless of nationality. Moreover, people who move from member countries of the European Economic Area can apply for health insurance from the day their legal residence is registered in Iceland.

The Icelandic Health Insurance (IHI) agency has been responsible for negotiating and purchasing services from independent OPT providers [17]. In 1999–2015, nearly all outpatient physical therapists had a contract with IHI in place. The insured clients paid a minimum fee for PT service, while the remaining cost was covered by the IHI. The fee (co-payment) for each OPT visit was fixed and decided in a regulation issued by the Ministry of Welfare. Old age pensioners (≥ 67 years), disability pensioners, people with serious diseases/disabilities, people with low income, and children (< 18 years) paid a lower fee. The OPT service (individual and group therapy) was reimbursed by the IHI and took place in special OPT clinics.

### Data sources and the study sample

The following Icelandic national electronic registries provided data for this study.

#### The Icelandic Health Insurance registry

Since 1999, IHI has kept electronic records. Our study is based on complete data from this national register with information on all clients who used OPT in a clinical setting and who were reimbursed by IHI from 1999 to 2015. Each of these 170,424 clients has a unique identification number in the IHI register. In our study each client was counted once per calendar year, but this same person was counted again if he/she used OPT in another calendar year. After summing up the cross-sectional data from 17 years we had a total of 516,161 client-years.

#### Statistics Iceland registry

Statistics Iceland (SI) collects, processes and disseminates data on the society, including complete information on all citizens’ age, sex and living conditions [23]. In this research we used data from the SI registry on the general population size by age and sex from 1999 to 2015, matching the end-of-year population size with information from IHI on their clients to estimate the fraction of each population age-sex subgroup that uses OPT services.

### Variables

Both the IHI registry and SI registry record complete data for the variables of sex and age. Our focus was on older adults (women and men ≥ 65 years) categorized into an age group variable: 65–74 years (young-old), 75–84 years (middle-old) and ≥ 85 years (oldest old).

### Statistical analysis

All analyses were performed in R. The precision of statistical estimates is reported as 95 % confidence intervals (95 % CI) and the level of significance was set at *p* < 0.05.

Simple counts and proportions were used to describe 1999 and 2015 older adults in the OPT client population and the total general Icelandic population, by all subgroups (age and sex). Rate Ratios (RR) and 95 % CI were used to identify whether these proportions changed between the two time-points (1999 and 2015) in both populations. To reduce the effect of random fluctuations from year to year the rate ratios were calculated based on the predictions of fitted regression models utilizing the data for all 17 years.

Log-linear regression models were used to determine whether there was an increase between years in the number of clients using OPT, adjusting for sex and age subgroups, and accounting for the general population growth [24]. For this analysis the two sources of data (IHI and SI) had to be combined in order to develop relative estimates of the ratio of OPT clients to the general population. More specifically, generalized linear models (GLMs) with Poisson response distributions and log links were used to estimate the number of older clients using OPT per calendar year [25]. The yearly trend in OPT client population numbers and general population numbers was estimated by including the current calendar year as a predictor variable and differences between age and sex subgroups within both populations were estimated by including interaction effects with the yearly trend [24]. To adjust for population growth and differences in the population sizes for different age and sex subgroups the natural logarithm of population count was used as an offset [25]. The potential for non-linear trends in the relative sizes within both populations was tested by comparing otherwise identical generalized additive models to the previously fitted GLMs and testing for statistically significant increases in model performance [24]. None of the null hypotheses of linear trends were rejected in favour of non-linear trends so the analysis proceeded using GLMs.

The beta-coefficient for year (from the GLM) was used to estimate the average yearly trend (average % change = (exp(beta)-1)*100) [25]. One full regression model was fitted containing the main effects of year as well as its interaction effect with age and sex subgroups. The results were then summarized into four models: (1) ≥ 65-year-old adults, (2) three age groups of older adults, (3) two sex groups, and (4) six age and sex groups of (women, men) X (65–74, 75–84, ≥ 85 years). The estimated yearly trend for each sub-model was calculated by averaging any necessary interaction effects [24].

## Results

**Fig. 1:**
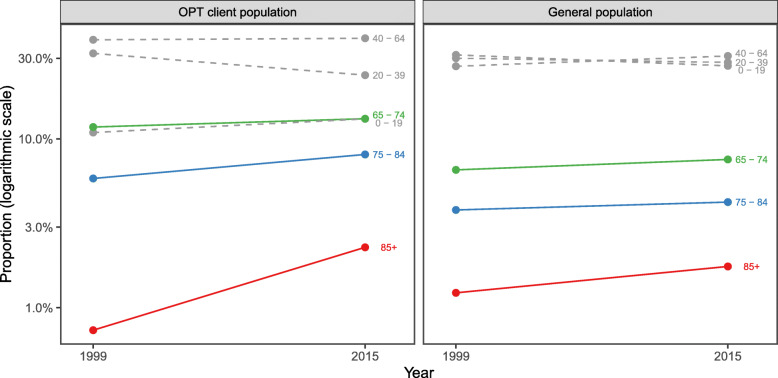
Proportions of different age groups in the outpatient physical therapy population and the general Icelandic population, in 1999 and 2015. Legend. OPT outpatient physical therapy; The proportions on the y-axes are on a logarithmic scale to accentuate the older age groups

Figure 1 presents an overview of the 1999 and 2015 proportions of older, middle aged, and younger age groups in the total population using OPT service in Iceland and in the general Icelandic population. From 1999 to 2015, the proportion of older adults (≥ 65 years) increased in both populations and this proportional increase was most prominent in the oldest old group (≥ 85 years).

On these two time-points (1999 and 2015) women were more prevalent among older OPT clients, yet within this older age-group of OPT clients the proportion of men increased from 36.1 % (1,599/4,432) to 38.1 % (3,955/10,350). In the general older Icelandic population, the proportion of men increased from 44.9 % (14,341/31,910) to 47.0 % (20,922/44,541) over these 17 years.

Table 1 reveals how proportions of older adults (≥ 65 years) in the OPT population and in the general population, changed between the two time-points 17 years apart. In 1999, older adults comprised 18.3 % of all OPT clients and in 2015 the proportion had increased to 23.5 %. Based on calculations of RR (Table 1), this means a 23 % increase in the proportion of older adults in the OPT population in 2015, compared to 1999. This increase in the proportion of older adults was significant in all subgroups (older age groups and sex), except among 65-74-year-old women. The increase was most marked (247 %) in ≥ 85-year-old men. In the general population, older adults comprised 11.6 % in 1999 and 13.5 % in 2015. Based on calculations of RR (Table 1), this means a 15 % increase in the proportion of older adults in the general population in 2015, compared to 1999. This increase was significant in all subgroups, except among 75-84-year-old women. The increase was most marked (58 %) in ≥ 85-year-old men.

**Table 1:** Proportions of older adults in the outpatient physical therapy population and the general total population^a^

	Outpatient Physical Therapy Population				General Total Population		
Sex	Age (years)	1999 n(%)	2015 n(%)	RR^**b**^ (95% CI)	1999 n(%)	2015 n(%)	RR^**b**^ (95% CI)
Overall:	Total (N)	24,188 (100%)	43,966 (100%)		275,712 (100%)	329,100 (100%)	
	65	4,432 (18.3%)	10,350 (23.5%)	1.23 (1.19-1.27)	31,910 (11.6%)	44,541 (13.5%)	1.15 (1.1-1.21)
	65 - 74	2,844 (11.8%)	5,792 (13.2%)	1.07 (1-1.15)	18,071 (6.6%)	24,865 (7.6%)	1.12 (1.05-1.19)
	75 - 84	1,410 (5.8%)	3,556 (8.1%)	1.26 (1.16-1.38)	10,461 (3.8%)	13,899 (4.2%)	1.11 (1.03-1.2)
	85+	178 (0.7%)	1,002 (2.3%)	3.41 (2.76-4.2)	3,378 (1.2%)	5,777 (1.8%)	1.49 (1.3-1.71)
Men:	Total (n)	9,205 (100%)	17,596 (100%)		138,086 (100%)	165,186 (100%)	
	65+	1,599 (17.4%)	3,955 (22.5%)	1.27 (1.21-1.33)	14,341 (10.4%)	20,922 (12.7%)	1.2 (1.14-1.27)
	65 - 74	1,035 (11.2%)	2,241 (12.7%)	1.14 (1.04-1.24)	8,668 (6.3%)	12,358 (7.5%)	1.16 (1.09-1.24)
	75 - 84	495 (5.4%)	1,344 (7.6%)	1.25 (1.12-1.40)	4,470 (3.2%)	6,413 (3.9%)	1.18 (1.09-1.29)
	85+	69 (0.7%)	370 (2.1%)	3.47 (2.65-4.54)	1,203 (0.9%)	2,151 (1.3%)	1.58 (1.35-1.86)
Women:	Total (n)	14,983 (100%)	26,370 (100%)		137,626 (100%)	163,914 (100%)	
	65+	2,833 (18.9%)	6,395 (24.3%)	1.22 (1.18-1.27)	17,569 (12.8%)	23,619 (14.4%)	1.12 (1.06-1.17)
	65 - 74	1,809 (12.1%)	3,551 (13.5%)	1.04 (0.98-1.11)	9,403 (6.8%)	12,507 (7.6%)	1.08 (1.01-1.15)
	75 - 84	915 (6.1%)	2,212 (8.4%)	1.28 (1.18-1.39)	5,991 (4.4%)	7,486 (4.6%)	1.06 (0.98-1.14)
	85+	109 (0.7%)	632 (2.4%)	3.4 (2.8-4.14)	2,175 (1.6%)	3,626 (2.2%)	1.45 (1.28-1.63)

^a^ Comparisons of 1999 and 2015, by age group and sex; ^b^ Rate ratios (RR) are calculated by applying log-linear regression models to data from all years from 1999 to 2015. Thus, they are not equal to simple RRs calculated using the percentages for the years 1999 and 2015 in this table but are more robust to sudden between-year leaps due to chance

**Fig. 2:**
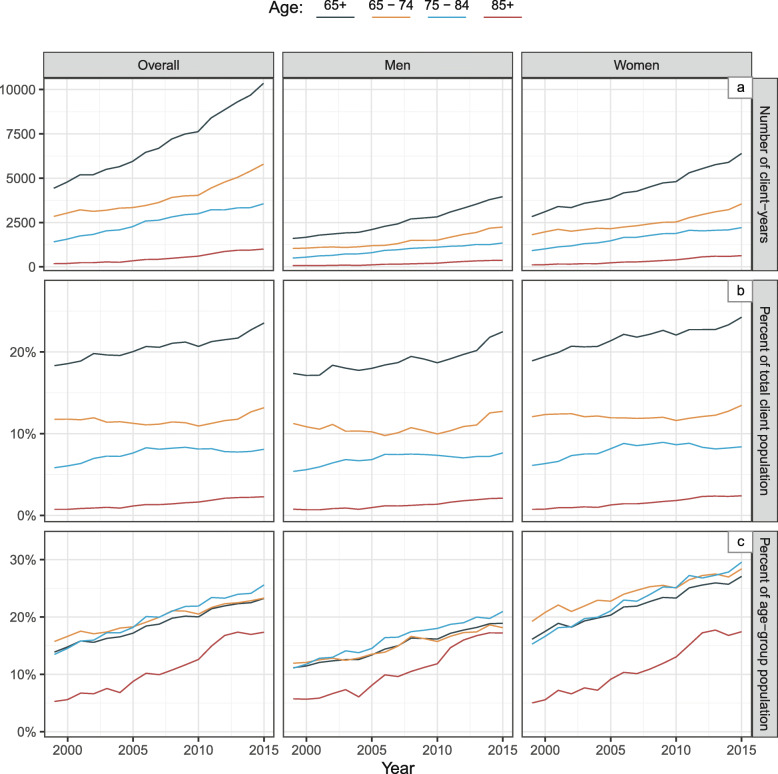
Yearly changes from 1999 to 2015 associated with older adults using outpatient physical therapy. Legend. The yearly changes from 1999 to 2015 in: **a** the absolute number of older adults who used outpatient physical therapy (OPT), **b** the percentage of older adults in the OPT client population (all ages), and **c** the percentage of the general older sub-population (≥ 65 years) who used OPT

Figure 2 describes the yearly trend from 1999 to 2015, associated with the older OPT clients. Figure 2a shows a yearly increase in the absolute number (client-years) of older OPT clients (older age groups; men and women). Figure 2b, reveals the yearly trend in the proportion of older adults (≥ 65 years) within the total OPT client population, reflecting how these proportions vary depending on calendar year (older age groups; men and women). Figure 2c presents a clear increase in the percentage of the general older population (age-group population) using OPT services each year (older age groups; men and women).

Linear modeling of all OPT clients’ data, revealed how the proportion of older adults in the OPT population increased linearly by 3.45 % for each year from 1999 to 2015 (Table 2, model a). This yearly change varied depending on older age group and sex (Table 2, models b-d) with the highest yearly increase in ≥ 85-year-old men, and no changes in 65-74-year-old women. Similarly, Table 2 reveals the yearly change in the rate of the older adults from the general population who used OPT (older age group; men and women).

**Table 2:** For each year from 1999 to 2015, linear increase was seen in the proportion of older adults within the OPT population and in the rate of older adults from the general population who used OPT

Sex	Age groups	Yearly % (95% CI)^**a**^ increase in:	
		the proportion of older adults in the OPT total population	the rate of older adults from the general total population who used OPT
**Model a**	65+	3.45 (3.05-3.85)	5.04 (4.56-5.52)
**Model b**	65 - 74	0.58 (0.25-0.92)	2.57 (2.18-2.96)
	75 - 84	1.63 (1.21-2.06)	3.96 (3.45-4.48)
	85+	8.46 (7.32-9.62)	8.87 (7.47-10.11)
**Model c**			
Men	65+	4.04 (3.51-4.57)	5.42 (4.79-6.06)
Women	65+	2.86 (2.43-3.30)	4.66 (4.15-5.17)
**Model d**			
Men	65 - 74	1.15 (0.68-1.62)	2.93 (2.40-3.48)
	75 - 84	2.21 (1.67-2.75)	4.34 (3.69-5.00)
	85+	9.11 (7.90-10.35)	9.18 (7.79-10.62)
Women	65 - 74	0.03 (-0.34-0.41)	2.21 (1.76-2.65)
	75 - 84	1.07 (0.61-1.52)	3.59 (3.05-4.14)
	85+	7.82 (6.68-8.98)	8.37 (7.07-9.70)

^a^Based on linear models describing the yearly changes from 1999 to 2015

## Discussion

This research was based on 17 years (1999–2015) of complete data from two national electronic registers. The results revealed that OPT clientele has been aging faster than the general population, and the clientele also reflects gradually increasing proportion of older men in the general population. Comparing the endpoints of the 17-year period, the proportion of older adults (≥ 65 years) within the total OPT clientele increased by 23 % (from 18.3 % to 1999 to 23.5 % in 2015) while in the general Icelandic population, the proportion of older adults increased by 15 % (from 11.6 % to 1999 to 13.5 % in 2015). Although women were in the majority among the older OPT clients, the proportion of older men was steadily increasing. Similar changes were seen in the general older population where the gender gap in life expectancy is decreasing. A more detailed analysis of each year within the 17-year period, revealed an overall 5 % yearly increase in the number of older adults from the general older Icelandic population who used OPT (accounting for population aging), and an overall 3.5 % yearly increase in the proportional contribution of older adults to the total OPT clientele. Both yearly trends in rate and proportions, varied depending on older age groups and sex, and the most prominent increase was among ≥ 85-year-old men.

Our results reflect how the overall growth in older adults who used OPT service exceeded what could be explained only by the growth in absolute number (and enlarging proportion) of older adults in the general total population. This increased OPT usage among the gradually enlarging general older population, was most pronounced for the ≥ 85 years age group and among men. This increase in older adults’ OPT usage, beyond population aging, may have various causes. First, older people are at the highest risk of all age groups of having poor health and of living with disabilities [11, 12] that may require PT services. Second, similar to many other societies, current Icelandic policies and practice promote aging in place and moving services from institutions into the community [17]. Therefore, older people with disabilities and poor health are at larger scale aging in their own homes and may be using an increased variety of outpatient services and forms of rehabilitation, including OPT. This explanation fits the fact that the observed rise in OPT usage was most profound in the ≥ 85-year-old group, which can be expected to include the frailest community dwelling individuals [26]. Only a few years ago this oldest group might have experienced prolonged hospital stays, inpatient PT and/or relocation into a nursing home. Third, the increasing number of older men seeking OPT may be directly associated with a decreasing gender gap in life expectancy in Iceland, as the life expectancy of men has been rising faster than that of women [16]. Finally, older generations are becoming increasingly empowered to optimize their functioning instead of passively accepting potentially manageable disabilities [13]. As the PT profession emphasizes movement and functioning [27], outpatient physical therapists are in a key position to meet these needs and those of current and future generations of community-dwelling adults.

Despite an absolute increase in clients of all age groups during the 17-year research period, the older adults entered OPT at a faster rate than people in other age groups. Therefore, the OPT client population is aging, as indicated by older adults increasing their share among OPT clients from 18.3 to 23.5 %. It is notable that women were more prevalent in the older OPT clientele than in the older general population. This could be due to the widely known old age gender gap in life expectancy, along with the old age gender disability gap reflecting women being more likely than men to age with and into disability [28–30]. Yet another explanation might be that men have been more reluctant to seek and receive help [31]. Importantly, despite women being in the majority among older OPT clients, the results also show a rise in the proportion of men (particularly the oldest old) using OPT. Perhaps this is an indication that there is a new generation of older men who are willing to seek appropriate health service to maximize their functioning and independence in old age. Our results resemble what was found in a Dutch study [32], which presented a constant increase in the use of OPT (all ages) associated with outpatient physical therapists becoming more accessible through direct access and client self-referral options. The researchers also revealed that from 2004 to 2010 the proportion of ≥ 60-year-old adults in the client population of OPT practices increased from 27.3 to 29.7 %, and the proportion of men in the client population (all ages) increased from 41 to 42.3 %. They concluded that population aging, and the increasing prevalence of chronic diseases contributed to the growing use of OPT among older people.

In many countries, outpatient physical therapists are in a key position to facilitate health and functioning among older adults, e.g. by routinely promoting physical activity [33] and including screenings for fall risk [34] on the initial examination for all new clients who have reached 65 years of age. Our findings confirm that there is an increasing need for geriatric expertise in the OPT field. Outpatient physical therapists must acknowledge that older adults make up a sizable and growing part of their clientele. Hence outpatient physical therapists should be competent in serving older adults and should be familiar with published evidence and guidelines aimed at physical therapists who work with aging clients. To ensure the delivery of best practice, outpatient physical therapists must know about aging-related physiological changes, multimorbidity, and contextual complexity [2–5, 35], and must be prepared to provide age-inclusive service, without old age stereotyping [36]. Raising the geriatric PT competency to an acceptable level is viable via multi-level educational approaches including;[3, 37] life-long learning, continuing education, entry-level PT programs, post-professional programs, and geriatric PT specialist certification.

Our results show that from 1999 to 2015, demographic changes (e.g. population aging) in the general Icelandic population followed very well what was predicted in 1999 [15]. The aging of the general population is predicted to continue [38], and the evidence indicates that we should respond to this prediction, be proactive, and prepare future outpatient physical therapists to work professionally and responsibly with aging clients. Outpatient physical therapists should build their future service planning on population statistics and demographic predictions for their service area, community, and society.

## Limitations and strengths

Limitations of this register-based study include that the variables are relatively simple (counts, age, sex), and there is a lack of background/contextual information. The results are based on number of individuals per year but not the volume of the OPT service (number of treatments) or the cost of service. Data are limited to one northern European country, and other countries and regions may have varying scenarios. While the focus of this research is on a whole nation, population growth and demographics can differ between geographical regions within each country [39]. For example, populations of rural and more remote areas commonly age faster than the more urban populations because of out-migration of younger people [40]. The strengths of our study include the following: a large sample size, great statistical power, use of real-world already existing data covering 17 years, and the data represent complete populations, which eliminates selection bias. The data were collected independently by national institutions, ensuring high quality of the variables we selected for the study, and creating a research database was relatively straightforward due to use of information technology. Our method of fitting a linear model to existing data is superior to calculating simple RRs between two years as in any given year there can be random fluctuations, either positive or negative, in the amount of OPT service, which will not be reflected in the RRs. Fitting a linear model to data from several years enabled us to smooth out these random fluctuations and discover the underlying trend. This can then be used to predict how clientele demographics will change in the future.

## Conclusion

 In reference to our study objectives, the results confirm that the OPT clientele has been reflecting changes in population demographics, where people are living longer, and in a context where life expectancy of men is approaching that of women. Therefore, these results support a routine use of demography to inform the planning of future OPT services. This is an encouragement for physical therapists all over the world to join or continue to follow the re-tooling wave of health care workforce for aging populations [1, 2]. Finally, our study is an example of how health-related and population-based registers can be used to provide important information for health services in general. Information which should be used to facilitate optimal planning of future health services, education, and research.

## Data Availability

The analysis was based on register data. Data from the registries are available for research projects which have been approved by the registries and the Icelandic National Bioethics Committee. Therefore, the authors are not allowed to share the data. For data requests contact sjukra@sjukra.is and information@statice.is.

## Abbreviations

PT: Physical Therapy
OPT: Outpatient Physical Therapy
IHI: Icelandic Health Insurance
SI: Statistics Iceland
RR: Rate Ratios
95 % CI: 95 % Confidence Interval
GLM: Generalized Linear Model
